# Cerebral venous thrombosis

**DOI:** 10.1590/0004-282X-ANP-2022-S108

**Published:** 2022-08-12

**Authors:** Anisio Adalio de Azevedo Moraes, Adriana Bastos Conforto

**Affiliations:** 1 Universidade de São Paulo, Faculdade de Medicina, Hospital das Clínicas, São Paulo SP, Brazil.

**Keywords:** Sinus Thrombosis, Intracranial, Decompressive Craniectomy, COVID-19, COVID-19 Vaccines., Trombose dos Seios Intracranianos, Craniectomia Descompressiva, COVID-19, Vacinas contra COVID-19

## Abstract

Cerebral venous sinus thrombosis (CVT) consists of partial or complete occlusion of a sinus or a cerebral vein. CVT represents 0.5-1% of all strokes and is more frequent in young women. This review discusses particular aspects of CVT diagnosis and management: decompressive craniectomy (DC), anticoagulation with direct oral anticoagulants (DOACs), CVT after coronavirus-disease 19 (COVID-19) and Vaccine-Induced Immune Thrombotic Thrombocytopenia (VITT).

## RESUMO

A trombose venosa cerebral (TVC) consiste na oclusão parcial ou completa de um seio ou de uma veia cerebral. A TVC representa 0,5-1% das doenças cerebrovasculares e é mais frequente em mulheres jovens. Esta revisão discute aspectos específicos do diagnóstico e do manejo da TVC: craniectomia descompressiva (DC), anticoagulação com anticoagulantes orais diretos (DOACs), TVC após infecção por coronavírus (COVID-19) e Trombocitopenia Trombótica Imune Induzida por Vacina (VITT).

## INTRODUCTION

Cerebral venous sinus thrombosis (CVT) consists of the partial or complete occlusion of a sinus or a cerebral vein. The earliest description of CVT dates from the first half of the 19^th^ century. Since then, it has become more and more recognized due to the widespread availability of advanced imaging techniques, such as CT venography, MR venography and digital subtraction angiography^1^. CVT represents 0.5-1% of all strokes and most often affects young women^2^. 

The most common occlusion sites are the transverse sinuses (44-73%), the superior sagittal sinus (39-62%), sigmoid sinus (40-47%), deep venous system (10.9%) and cortical veins (3.7-17.1%)^3^. The clinical presentation of CVT is variable. Headache is usually the most common symptom (88.8%), followed by seizures (39.3%) and paresis (37.2%). It may also present with other focal neurologic deficits or altered mental status^4^. Intracranial hemorrhage occurs in 30-40% of the patients^5^. Management is based on anticoagulation, even in most of the patients with hemorrhagic lesions. Heparin in the acute phase, followed by vitamin K antagonists (VKA), is the standard approach. Direct oral anticoagulants (DOACs) have been discussed as potential options^6^.

CVT has a good prognosis compared to other types of cerebrovascular disorders. The *Prognosis of Cerebral Vein and Dural Sinus Thrombosis: Results of the International Study on Cerebral Vein and Dural Sinus Thrombosis* (ISCVT) study identified factors associated with poor outcomes: male gender, age above 37 years, GCS <9 on admission, deep venous system thrombosis, hemorrhagic lesions, central nervous system infection and malignancy^4^. The CVT risk score^7^ is aimed to predict outcome after six months of CVT and it was developed based on ISCVT data. It ranges from 0 to 9 and, with a cut-off of 3 points, it has a sensitivity of 95.5% and a specificity of 13.6%, being useful to identify high-risk patients.

About 15% of patients with CVT die or remain disabled^2^ ^,^ ^6^. The main cause of death in the acute phase is transtentorial herniation secondary to a large hemorrhagic lesion or to diffuse brain edema^8^. Good outcomes were reported in most of the patients treated with decompressive craniectomy (DC) after CVT in observational studies^5^ ^,^ ^9^ ^-^ ^13^.

 Permanent (e.g., genetic thrombophilia) and transient (e.g., puerperium, infections, oral contraceptives) risk factors can increase the risk of CVT^8^. Coronavirus disease (COVID-19) and Vaccine-Induced Immune Thrombotic Thrombocytopenia (VITT) have emerged as risk factors after the onset of the pandemic^14^.

 We reviewed specific aspects of CVT diagnosis and management: decompressive craniectomy (DC), anticoagulation with DOACs, CVT after COVID-19 and VITT.

## DECOMPRESSIVE CRANIECTOMY

 Randomized clinical trials showed that DC decreases mortality and major disability in selected patients with malignant middle cerebral artery infarction^15^. Until now, no randomized clinical trials were performed to assess the benefits of DC in patients with CVT. Information is limited to case series and systematic reviews^5^ ^,^ ^9^ ^,^ ^16^. Here, we discuss some questions about DC in CVT. 

### Who should undergo DC, and when?

Coutinho et al.^10^ reported three patients treated with DC and reviewed 10 additional cases reported in the literature. Patients underwent surgery after presenting third nerve palsy or deterioration on the Glasgow coma score (GCS) caused by large cerebral edema (midline shift of 9-15mm) associated with hemorrhagic lesions. In this series, 11/13 patients had a good outcome defined as a modified Rankin scale (mRS)^17^ ≦3. Théaudin et al.^11^ reported a series of 12 patients with CVT and decreased consciousness with dilated pupils or radiological signs of transtentorial herniation (median midline shift of 12mm). Eight were treated with DC and four were not. All patients who did not undergo craniectomy died. Seven of 8 patients who underwent craniectomy survived. In the last follow-up (median, 23.1 months) 6/7 survivors presented no disability, with scores of 0 or 1 in the mRS. Ferro et al.^9^ reviewed 69 cases of CVT treated with DC. Only 12 (17.4%) had an unfavorable outcome (mRS 5-6) at last follow-up (median, 12 months). Aaron et al.^12^ reported 44 patients with or without hemorrhagic lesions who underwent DC. The mortality rate was 20% (9/44). mRS≦2 was achieved in 27/35 (77.1%) of the survivors. Zhang et al.^5^ analyzed 58 patients with hemorrhagic CVT who had undergone DC and 33 (56.9%) achieved a favorable outcome (mRS 0-2) at the 6-month follow-up. 

Similar results were described in developing countries. Vivakaran et al.^18^ reported that 26/34 (76.5%) of patients in India had a favorable functional outcome (Glasgow outcome scale^19^ 4-5) after DC. All patients had hemorrhagic infarctions with midline shift (mean, 10.6mm). A retrospective series from Pakistan^13^ described good functional outcome (mRS 0-2) in 4/7 (57.1%) of the operated patients.

Final results from *Decompressive Neurosurgery for Patients with Cerebral Venous Thrombosis: A Prospective Multicenter Registry* (DECOMPRESS2) cohort were presented at the 7^th^ European Stroke Organization Conference. Despite a severe clinical condition at baseline, patients kept improving even after 6 months of follow-up. About 2/3 of CVT patients were alive, and 1/3 were independent (mRS 0-2) one year after the surgery, consisting of DC with or without hematoma drainage. DC was considered a worthwhile procedure by more than 80% of patients and caregivers^20^.

In summary, emergency decompressive surgery is a life-saving intervention in patients with mass effect and midline shift who worsen despite adequate anticoagulation. This indication is supported by an AHA/ASA statement^21^ and by the ESO-EAN guideline^22^. There is a scarcity of data regarding DC in patients with CVT because of logical ethical issues, leading to potential bias due to non-uniform criteria to indicate this procedure. Some authors have selected patients by the radiologic status, such as the presence of hemorrhagic lesions or the extent of midline shift. Others prefer to select patients by clinical status, defined by GCS or pupillary reaction. Decisions should be individualized. According to the results of DECOMPRESS-2, outcomes in patients with CVT treated with DC, with or without hematoma drainage, are worse than previously believed based on systematic reviews, but are still two times better than in patients with malignant middle cerebral artery infarcts submitted to DC^15^.

When it comes to the optimal timing of surgery, there are conflicting opinions. Vivakaran et al.^18^ concluded that the duration of coma or the time between neurological deterioration and surgery was not of a significant predictor of surgical outcome. On the other hand, Aaron et al.^12^ reported that surgery delayed by >12 h is a significant predictor of mortality. The time from symptom onset to surgery was also considered a potential risk factor for poor outcomes by Zhang et al.^5^. Raza et al.^13^ proposed that early intervention, when GCS scores start to decrease but the pupillary response is still preserved, may be the best moment for surgery. Despite divergent data, we believe that early surgery, before irreversible damage occurs due to herniation, is preferable. However, there is a need for more evidence about the optimal moment for DC in CVT.

### How should DC be performed?

Depending on the location of the lesion, craniectomy may be performed by different approaches^9^. Bilateral frontal lobe involvement, usually secondary to anterior superior sagittal sinus thrombosis, has rarely been treated with bifrontal craniectomies, while posterior craniectomies are required to treat posterior fossa lesions. 

Dural opening is a standard approach^5^ ^.^ ^10^ ^-^ ^12^ ^,^ ^18^ ^,^ ^23^. Hematoma evacuation is also not mandatory, being reserved for large hematoma volumes with mass effect, and is a matter of debate. The brain flap is often replaced after 3-6 months, after the brain swelling resolves^2^.

### When should anticoagulation be restarted?

AHA/ASA and ESO-EAN guidelines do not provide any specific recommendation about the optimal time to return anticoagulation after surgery^21^ ^,^ ^22^. Furthermore, there are no specific guidelines about the timing of anticoagulation for CVT after hemicraniectomy^24^. Anticoagulation has been restarted between 12 hours and 8 days after surgery and must be individualized^2^ ^,^ ^13^.

## DOACs AND CVT

Anticoagulation is the standard approach to treat CVT, and most of the time, is performed even when intracranial hemorrhage is present. Heparin followed by VKA is recommended by current guidelines^21^ ^,^ ^22^. This routine was initially derived from the management of venous thromboembolism (VTE) of other more usual sites, such as deep venous thrombosis (DVT), albeit there has always been concern about the risk of increase in size of intracranial hemorrhagic lesions. Over time, randomized controlled trials and meta-analyses have endorsed the safety and effectiveness of anticoagulation with heparin followed by VKA in CVT^25^ ^-^ ^27^. 

More recently, direct oral anticoagulants (DOACs) have emerged as alternatives to warfarin for treatment of patients with VTE^28^ ^,^ ^29^ and for secondary embolism prevention in non-valvular atrial fibrillation (AF)^30^ ^,^ ^31^. The safety and efficacy of DOACs have been well demonstrated in AF and VTE populations^32^ ^-^ ^36^. Anticoagulant effects of DOAC do not require monitoring, and the potential for interactions with other drugs is lower for DOACs than for warfarin. The performance of clinical trials that compare DOACs and warfarin for prevention of CVT recurrence is challenging given the relative low frequency of CVT in the general population. 

The *Safety and Efficacy of Dabigatran Etexilate vs Dose-Adjusted Warfarin in Patients With Cerebral Venous Thrombosis - A Randomized Clinical Trial* (RE-SPECT-CVT)^6^ is the only published randomized controlled trial that compared outcomes between patients treated with dabigatran, a thrombin inhibitor, and VKA. The study included patients aged 18-79 years, clinically stable after receiving acute CVT treatment. The exclusion criteria were: CVT associated with central nervous system infection or major head trauma, major bleeding in the previous six months, malignancy, and creatinine clearance level less than 30ml/min. In this trial, 120 patients were randomized to DOAC or warfarin after 5-15 days of the initial acute treatment with unfractionated or low-molecular-weight heparin. In the warfarin group, the target international normalized ratio (INR) goal was 2-3. The dose of dabigatran was 150mg twice daily. The primary outcome was the composite of major bleeding or VTE (recurrent CVT, DVT, pulmonary embolism (PE) or splanchnic vein thrombosis). Patients were followed up to 25 weeks. No recurrence of VTE was observed in either group. One major hemorrhage occurred in the dabigatran group, and two, in the warfarin group. Despite the same risk of recurrence and similar few major bleeding events, it was not possible to demonstrate the noninferiority or superiority of either treatment because of the limited simple size and the low rates of VTE recurrence and hemorrhagic complications. 

Connor et al.^37^ assessed the use of DOAC in children with venous thrombosis. One hundred and seventeen children were randomized in a 2:1 ratio to receive either open-label rivaroxaban or standard VKA anticoagulation. The inclusion criteria were children with CVT already being treated with unfractionated heparin, low-molecular-weight heparin, or fondaparinux. The major exclusion criteria were active bleeding or a high risk of bleeding, and an estimated glomerular filtration rate <30 mL/min per 1.73 m2. The primary outcome was symptomatic recurrent VTE (DVT; PE; CVT; or jugular, caval, renal, portal vein, or catheter-related thrombosis). The primary outcome was not observed in any of the patients in the rivaroxaban group and was reported in one subject in the warfarin group. Also, none of the children in the rivaroxaban group, and one in the warfarin group, presented a major hemorrhage. A similar effect on clot resolution was demonstrated in the rivaroxaban group, compared to the warfarin group. 

Nepal et al.^38^ published a systematic review and meta-analysis to assess the efficacy and safety of long-term use of DOAC for CVT treatment. In a comparative analysis of DOAC versus VKA, 295 patients were included in the DOAC group and 470 in the VKA group. No significant difference was observed between the rates of CVT in the two groups (1.03% vs. 1.06%, respectively; I²=0%). 

The *Direct Oral Anticoagulants Versus Warfarin in the Treatment of Cerebral Venous Thrombosis (ACTION-CVT): A Multicenter International Study* was a large multicenter international retrospective cohort of patients diagnosed with CVT^39^. A total of 845 patients were included in the study between 2015 and 2020. Patients were excluded if a specific anticoagulation strategy (DOAC or warfarin) was preferred. For instance, in CVT associated with antiphospholipid antibody syndrome, warfarin is the drug of choice^40^. In ACTION-CVT, 33% (279) of the patients received DOAC only, 51.8% (438) received warfarin only, and 15.1% (128) received both treatments at different times. Among patients who received DOAC, the majority (66.6%) received apixaban. The primary outcome, recurrent venous thrombosis (VTE or CVT), occurred in 5.26 per 100 patient-years in patients treated with DOACs versus 5.87 per 100 patient-years in subjects treated with warfarin (hazard ratio, 0.86 [95% CI, 0.47-1.56]; P=0.61). Treatment with DOAC was associated with similar risks of death, as well as with rates of recanalization, compared to treatment with warfarin. Major hemorrhage was significantly less frequent in patients treated with DOACs than in those treated with warfarin (HR=0.35; 95% CI=0.15-0.82; P=0.02). The conclusions from the study are limited by the potential of bias, considering the retrospective design. Also, the number of patients treated with DOACs was smaller than the number of subjects treated with warfarin. 

These preliminary studies suggest that DOACs may be safely administered and that the risk of recurrence on DOACs or warfarin is low in patients who fulfill eligibility criteria for RE-SPECT-CVT and ACTION-CVT. Two ongoing studies, the observational DOAC-CVT (*Direct Oral Anticoagulants in the Treatment of Cerebral Venous Thrombosis*, NCT04660747), and the randomized SECRET phase 2 clinical trial (*Study of Rivaroxaban for Cerebral Venous Thrombosis,* NCT03178864), are expected to provide more information about the use of DOACs for prevention of thrombotic events after CVT. 

## CVT IN PATIENTS WITH COVID-19

 SARS-CoV-2 infection may be associated with vascular and neurological manifestations^41^. Up to 1/3 of critically ill patients with COVID-19 may present thromboembolic phenomena^42^. Hughes et al.^43^ reported the first case of CVT in a patient with COVID and since then, several cases of CVT in COVID have been reported. In a retrospective cohort study with over 500,000 COVID-19 cases, the incidence of CVT within two weeks after the diagnosis of COVID-19 was 42.8 per million people, about three times higher than before the pandemic^1^ ^,^ ^8^.

Baldini et al.^44^ published a systematic review and meta-analysis of CVT in patients with SARS-CoV-2 infection, including a total of 57 CVT cases from 28 different studies. The mean age was 53.5 years and gender was equally distributed. CVT preceded the diagnosis of SARS-CoV-2 infection in only four cases. The interval from the onset of COVID-19 respiratory symptoms to the symptoms of CVT ranged from 0 to 47 days. None of the patients had thrombophilia or history of prior episodes of venous thrombosis. Five women were taking oral contraceptives, two subjects had solid tumors, one had polycythemia, another one had a traumatic skull fracture and one child had concomitant tuberculous meningitis. The sites of thrombosis were reported in 43 patients: the transverse sinus was involved in 65%, the sigmoid sinus, in 47%, the superior sagittal sinus, in 44% and the straight sinus, in 21%. The involvement of multiple venous sinuses was frequent. Imaging showed hemorrhagic lesions in 42% of the cases. Fibrinogen levels were abnormal in 54.5% of the patients (mean, 490.8 ± 112.9 mg/dl) and d-dimer levels, in all but two cases (mean, 7812 ± 15 ng/ml). Anticoagulants were administered to 95% of the patients. The in-hospital death rate was 40%.

Multiple mechanisms may play a role in the risk of thrombosis in patients with CVT and COVID-19. An important mechanism is the linkage of the coronavirus to the angiotensin converting enzyme-2 receptor expressed in vascular endothelial cells, leading to direct endothelial damage. All components of the Virchow’s triad (endothelial dysfunction, altered flow dynamic and hypercoagulable state) may be present^45^.

Headache, focal motor deficits and seizures are the main symptoms, just like CVT in patients without COVID-19^41^ ^,^ ^44^. Therefore, patients presenting with these symptoms should prompt investigation of cerebrovascular events^46^. Anticoagulation is the standard treatment approach^45^. Different studies described higher death rates in patients with CVT and COVID-19 compared with those without COVID-19. This finding may be related to a more aggressive hypercoagulable state or to the severity of COVID-19 itself^44^ ^,^ ^46^ ^,^ ^47^.

## VACCINE-INDUCED IMMUNE THROMBOTIC THROMBOCYTOPENIA (VITT)

After vaccination against SARS-CoV-2 was initiated in Europe and North America, a new prothrombotic syndrome, VITT, was associated with the adenovirus vector-based vaccines ChAdOx1 CoV-19 vaccine (AstraZeneca/Oxford)^14^ ^,^ ^48^ and the Ad26.COV2.S COVID-19 vaccine (Janssen/Johnson & Johnson)^49^. VITT consistently presents with thrombosis at unusual sites, such as CVT and portal vein thrombosis, associated with thrombocytopenia and high levels of D-Dimer. These clinical features resemble heparin-induced thrombocytopenia (HIT)^50^, except for the absence of recent heparin exposure. The identification of anti-platelet factor 4 (PF4)-heparin complex antibodies in VITT patients’ serum^51^ suggests that similar mechanisms underlie HIT and VITT. 

Bilotta et al.^52^ published a systematic review of VITT, including a total of 58 subjects vaccinated with the ChAdOx1 CoV-19 vaccine (AstraZeneca/Oxford) reported in seven studies. Their ages ranged from 21 to 77 years and most of the subjects were women. The mean interval between vaccination and admission ranged from seven to 16 days. Headache was the most common symptom on admission. CVT was the most common frequent type of thrombosis. The mean platelet count ranged from 5,000/µL to 49,200/µL. Positive anti-PF4 antibody tests were present in 93% of the patients. Death was the most common outcome, except in one study that reported a 70% survival rate.

Maryam et al.^53^ published a systematic review of CVT after COVID-19 vaccination. A total of 54 patients from 14 studies were included, 41 after receiving the AstraZeneca/Oxford vaccine and 13, after the Janssen/Johnson & Johnson vaccine. There was a female predominance (36/45 with available data about gender). Symptom onset after vaccination ranged from four to 19 days. Headache was the most frequent presenting symptom. Other sites of thrombosis were deep, splanchnic, portal, iliofemoral vein or internal jugular veins; lung (PE) and bowel. The mean platelet count was 39,000/µL, ranging between 5,000/µL and 127,000/µL. The PF4 IgG assay was positive in 27 patients and D-dimer levels were increased in 35 patients. A total of 21 (38.8%) patients died.

In summary, VITT is associated with a high risk of death. Thereafter, a team of experts including neurologists, hematologists and intensive care specialists should assist patients with this condition. Acute management of VITT is based on the use of non-heparin anticoagulants, including factor Xa inhibitors (apixaban, rivaroxaban, edoxaban, fondaparinux, danaparoid) or direct thrombin inhibitors (argatroban, bivalirudin or dabigatran), associated with intravenous immunoglobulin (IVIG) at a dose of 1g/Kg daily for two days, irrespective of the degree of thrombocytopenia^53^ ^-^ ^56^. Therapeutic plasma exchange (TPE) may be an alternative to IVIG in progressive cases and should be considered in cases of VITT with CVT^55^. Steroids should be considered in case of unavailability of IVIG and TPE^53^. Fibrinogen levels should be kept above 150mg/dL with cryoprecipitate or fibrinogen concentrate. Rituximab may play a role for patients who do not respond to IVIG and TPE treatment^55^.

Salih et al.^57^ reported 11 patients presenting initially with headache associated with thrombocytopenia, high D-dimer levels and high levels of anti-PF4-heparin IgG antibodies after 5-18 days of ChAdOx1 nCoV-19 (AstraZeneca) vaccination, with no CVT or other types of thrombosis. None of the seven patients who received treatment with anticoagulation or immunosuppression within five days of headache onset developed a thrombotic complication. However, 4/5 patients who were not initially treated, later presented thrombosis (CVT, PE or splanchnic vein thrombosis). The authors considered this initial presentation as representing a “pre-VITT syndrome” and suggested that anticoagulation may be considered to prevent thrombotic complications.

 After the recognition of VITT, vaccination with adenovirus vector-based vaccines was interrupted in the United States and European countries^58^. However, the risk of death or thrombotic complications after COVID-19 is higher than the risk of presenting VITT after vaccination with AstraZeneca or Johnson & Johnson vaccines^53^. For those who have developed VITT after an adenovirus vector-based vaccine, a mRNA vaccine may be considered for the booster shot^55^.

 In conclusion, DC benefits selected patients with CVT and DOACs have emerged as potentially safe alternatives to VKA. Two new risk factors for CVT have been identified: COVID-19 and adenovirus vaccines against SARS-CoV-2. Recognizing these factors is crucial for proper diagnosis and treatment. 
